# Buffalo at the A. M. A. Meeting

**Published:** 1911-06

**Authors:** 


### Buffalo at the A. M. A. Meeting

THE program for the meeting of the American Medical Association at Los Angeles in June has been published in the *Journal of the American Medical Association* and contains the names of few Buffalo physicians. Allen A. Jones is chairman of the section of practice of medicine. He is the only Buffalonian occupying official position. F. Park Lewis will read a paper on Intraocular Neoplasms, with report of five cases before the section on ophthalmology and Lucien Howe, before the same section, will make the report of the committee on collective investigation concerning the ocular muscles. Before the section on preventive medicine and public health, F. Park Lewis will present a paper on Prevention of Ophthalmia Neonatorum. George E. Fell will present before the section, papers on Control of Drinking Water and the Advisability of Government Medical Control of the Hygienic Factors of Waters of the Great Lakes.

Four papers presented by two men, a committee report by another and the holding of a section chairmanship is the total of Buffalo's representation at this meeting. This seems a rather attentuated display for a city of the size and medical importance of Buffalo and an effort should be made for a better showing at subsequent meetings. There is every indication that other cities through their societies and associations, make definite effort for representation at national meetings and the profession in Buffalo should take kindly to the advice contained in Dr. McCormack's recent heart to heart talk to the profession and "stand together."

